# Small-volume resuscitation with hyperoncotic albumin: a systematic review of randomized clinical trials

**DOI:** 10.1186/cc6812

**Published:** 2008-03-04

**Authors:** Matthias Jacob, Daniel Chappell, Peter Conzen, Mahlon M Wilkes, Bernhard F Becker, Markus Rehm

**Affiliations:** 1 Klinik für Anästhesiologie, Ludwig-Maximilians-Universität, Klinikum Grosshadern, Nussbaumstrasse 20, D-80336 Munich, Germany; 2 Hygeia Associates, 17988 Brewer Road, Grass Valley, California 95949, USA; 3 Physiologisches Institut – Vegetative Physiologie, Ludwig-Maximilians-Universität, Schillerstrasse 44, D-80336 Munich, Germany

## Abstract

**Background:**

Small-volume resuscitation can rapidly correct hypovolemia. Hyperoncotic albumin solutions, long in clinical use, are suitable for small-volume resuscitation; however, their clinical benefits remain uncertain.

**Methods:**

Randomized clinical trials comparing hyperoncotic albumin with a control regimen for volume expansion were sought by multiple methods, including computer searches of bibliographic databases, perusal of reference lists, and manual searching. Major findings were qualitatively summarized. In addition, a quantitative meta-analysis was performed on available survival data.

**Results:**

In all, 25 randomized clinical trials with a total of 1,485 patients were included. In surgery, hyperoncotic albumin preserved renal function and reduced intestinal edema compared with control fluids. In trauma and sepsis, cardiac index and oxygenation were higher after administration of hydroxyethyl starch than hyperoncotic albumin. Improved treatment response and renal function, shorter hospital stay and lower costs of care were reported in patients with liver disease receiving hyperoncotic albumin. Edema and morbidity were decreased in high-risk neonates after hyperoncotic albumin administration. Disability was reduced by therapy with hyperoncotic albumin in brain injury. There was no evidence of deleterious effects attributable to hyperoncotic albumin. Survival was unaffected by hyperoncotic albumin (pooled relative risk, 0.95; 95% confidence interval 0.78 to 1.17).

**Conclusion:**

In some clinical indications, randomized trial evidence has suggested certain benefits of hyperoncotic albumin such as reductions in morbidity, renal impairment and edema. However, further clinical trials are needed, particularly in surgery, trauma and sepsis.

## Introduction

The advantages of small-volume resuscitation in rapidly correcting hypovolemia and interrupting the pathological processes leading to multi-organ failure and other poor outcomes are well recognized [1]. The use of hyperosmolar solutions containing artificial colloid for small-volume resuscitation has been described previously [2]. Another fluid type suitable for small-volume resuscitation is hyperoncotic (20 to 25%) albumin. Indeed, the original form of albumin developed in the early 1940s for resuscitation of combat casualties was a hyperoncotic 25% solution, designed for portability [3]. Currently, 4 to 5% albumin solutions are also widely employed for volume expansion. Whereas 4 to 5% albumin expands intravascular volume by approximately 80% of the administered volume [4-15], the corresponding effect of 20% albumin averages 210% [4,16-18] and 25% albumin 260% [4,19-21]. Consequently, hyperoncotic albumin can accomplish the same volume expansion effect as 4 to 5% albumin using only roughly one-third of the administered volume, thus diminishing the time needed to attain the desired expansion of the intravascular space. The disproportion in administered volume is far greater vs crystalloid, since, for example, the required volume of Ringer's lactate (RL) was fourfold that of 5% albumin to achieve the same hemodynamic endpoints in a randomized trial of patients with multiple trauma and shock [22]. Furthermore, the effect of hyperoncotic albumin is relatively long lasting, with at least two-thirds of the initial volume expansion effect persisting at 6–8 h after infusion [19,21].

Hyperoncotic albumin also possesses the capacity to draw interstitial fluid into the intravascular space, in accord with the Starling fluid equilibrium equation [23]. Thus, undesirable edema may be reduced [24].

Nonetheless, it has remained unclear whether the properties of hyperoncotic albumin can translate into demonstrable clinical benefits. The only large-scale randomized trial of outcomes after albumin administration, the Saline versus Albumin Fluid Evaluation (SAFE) trial [25], compared 4% albumin with normal saline for management of hypovolemia, and no difference in survival was detected. Systematic reviews of mortality, morbidity and other endpoints thus far have not discriminated between 4 to 5% and 20 to 25% solutions of albumin [26-30]. The multi-center non-randomized observational Sepsis Occurrence in Acutely ill Patients (SOAP) study also did not differentiate between types of albumin solutions [31].

The present systematic review of randomized clinical trials is focused exclusively on hyperoncotic albumin solutions. A qualitative critical appraisal of the randomized trial evidence is presented. Additionally, a quantitative meta-analysis of survival after hyperoncotic albumin administration is reported.

## Methods

### Objectives

This systematic review of randomized clinical trials was undertaken to determine whether hyperoncotic albumin consistently differs from control regimens in its effects upon clinically relevant endpoints such as morbidity, major organ function, length of stay and cost of care in acutely ill patients. Such endpoints are often not defined, assessed and reported in a consistent and standardized manner, so qualitative summarization is more appropriate than quantitative combination of results across trials. A secondary objective was to evaluate the effect of hyperoncotic albumin on survival by quantitative meta-analysis.

### Inclusion criteria

All randomized clinical trials comparing hyperoncotic albumin with a control regimen for volume expansion in acutely ill patients were eligible for inclusion. Both parallel-group and crossover study designs were acceptable. Randomized trials focusing on other uses of albumin such as treatment of hyperbilirubinemia, extracorporeal albumin dialysis or prevention of ovarian hyperstimulation syndrome were excluded. Also excluded were randomized trials of albumin as an adjunct to paracentesis or for correction of hypoalbuminemia, both of which have been evaluated in previous systematic reviews [28,29].

### Identification of studies

Randomized clinical trials fulfilling the inclusion criteria for the systematic review were identified by multiple methods, including computer searches of the Medline and EMBASE bibliographic databases and the Cochrane Library. Search terms included hyperoncotic albumin, resuscitation, hypovolemia, surgery, trauma, sepsis, liver diseases, intensive care, neonatal, brain injuries, nephrotic syndrome and randomized controlled trials. Studies were also sought through examination of reference lists and manual searching of Index Medicus and specialty journals. No restrictions on time period or language of publication were applied.

### Data extraction

Data on numbers of patients randomized, clinical indication, hyperoncotic albumin and control regimen, major findings and deaths were extracted from the randomized clinical trial reports and used to populate a relational database. The participating investigators, time periods and study subject characteristics were closely examined to avoid inclusion of duplicate datasets appearing in multiple publications and to ensure the most complete possible dataset. Deaths were recorded on an intent-to-treat basis. Unpublished survival data and clarification regarding study design features were sought as needed via direct inquiries with the randomized trial investigators.

### Quality assessment

Trial quality was evaluated by the criteria of blinding and allocation concealment. On the basis of the procedures employed in the study, allocation concealment was classified as adequate, inadequate or unclear [32].

### Statistical analysis

R version 2.4.1 (The R Foundation for Statistical Computing, Vienna, Austria), Stata 9.1 (Stata Corp., College Station, TX, USA) and SPSS 11.5 (SPSS Inc., Chicago, IL, USA) statistical software was used for analyses. The attributes of the included trials were summarized by descriptive statistics, i.e., the median and interquartile range (IQR).

The primary endpoint for the quantitative meta-analysis of survival was the relative risk (RR) of death. The 95% confidence interval (CI) of RR was also calculated. RR can only be calculated when at least one death has been observed; hence, trials with no deaths did not enter the survival meta-analysis. Deaths in crossover trials were also excluded from the meta-analysis, since in such trials the same patients are exposed to both the albumin and control regimens and the attributability of death to one regimen or the other is uncertain. Pooled RR was estimated under a fixed-effects model. Publication bias was assessed by the method of Egger *et al. *[34]. The statistical power of the meta-analysis was calculated as 1 - Φ (C_α/2 _- λ) + Φ (-C_α/2 _- λ), where Φ = the standard normal cumulative distribution function, C_α/2 _= the standard normal critical value for a two-sided test at the 0.05 α level, and λ = (ln(pooled RR) - 1)/(pooled standard error) [34].

## Results

### Included trials

**Figure 1:**
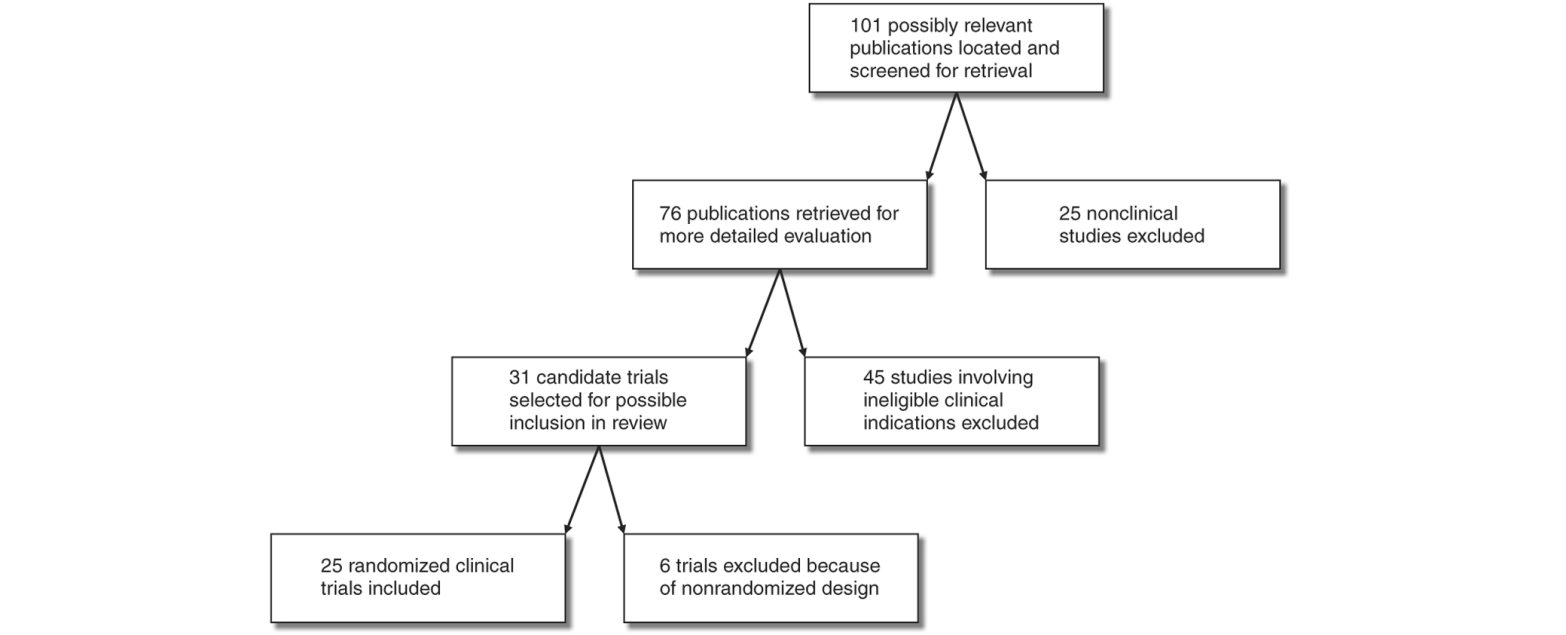
Selection process for randomized clinical trials.

A total of 25 randomized clinical trials [35-59] with an aggregate of 1,485 patients were included in the review (Figure 1). The median number of patients per trial was 30 (IQR, 18–58). In all, 21 of the trials (84%) had been published from 1990 onwards. Parallel groups were compared in 21 trials, and a crossover design was employed in the remaining 4. The control regimen consisted of another colloid, predominantly hydroxyethyl starch (HES), in nine trials (36%), crystalloid in four (16%), no albumin in six (24%) and lower-dose albumin in one (4%). Two separate control regimens composed of another colloid and either crystalloid or no albumin were administered in five trials (20%).

In five trials, the effects of hyperoncotic albumin were separately investigated among patients with either trauma or sepsis [44-46,48,49]. Randomized comparisons involving surgery and trauma are summarized in Table 1, sepsis in Table 2 and other indications in Table 3. The other indications were comprised of liver disease, high-risk neonates, brain injury, intradialytic hypotension and nephrotic syndrome. The trials of high-risk neonates involved premature infants with risk factors such as low birth weight, respiratory dysfunction or brain injury.

**Table 1:** Randomized clinical trials of hyperoncotic albumin in surgery and trauma

**Trial**	**n**	**Indication**	**Regimen**	**Results**
Cardiac surgery				
Boldt *et al.*, 1986 [37]	55	Coronary artery bypass grafting	300 ml 20% albumin intraoperatively after bypass vs 500 ml 3% HES 200/0.5 vs 500 ml 3.5% gelatin vs no additional volume	Post-bypass COP rebound greater in albumin than other groups (p < 0.05)
Boldt *et al.*, 1993 [41]	30	Cardiac defect repair in children < 3 years old	20% albumin vs 6% HES 200/0.5 to stabilize hemodynamics before bypass	On-bypass urine output in HES group lower by 57% than that of albumin group (p < 0.05)
Magder and Lagonidis, 1999 [52]	28	Stable patients after cardiac bypass surgery	100 ml 25% albumin vs saline to increase right atrial pressure by 2 mm Hg	Greater increase in cardiac output among hyperoncotic albumin recipients, suggesting an inotropic effect
Non-cardiac surgery				
Zetterström and Hedstrand, 1981 [36]	30	Elective major abdominal surgery	300–400 ml 20% albumin on operation day, 200 ml on next day and 100 ml/day for subsequent 3 days vs no albumin	In albumin recipients COP significantly closer to preoperative level on postoperative days 2–6
Prien *et al.*, 1990 [39]	18	Abdominal surgery	20% albumin vs 10% HES 200/0.5 vs Ringer's lactate to maintain preoperative CVP	Significantly lower intraoperative intestinal edema after albumin compared with either HES or Ringer's lactate; impaired coagulation in HES recipients
Trauma				
Boldt *et al.*, 1995 [44]	30	Trauma of ISS > 15	20% albumin vs 10% HES 200/0.5 to 12–16 mm Hg target CVP, PCWP or both	No between-group differences in daily profiles of plasma thrombomodulin, proteins C and S and thrombin-antithrombin III
Boldt *et al.*, 1996 [45]	30	Trauma of ISS between 15 and 30	20% albumin vs 10% HES 200/0.5 to 12–18 mm Hg target PCWP	HES 200/0.5 but not albumin increased cardiac index, PaO_2_/FiO_2_, DO_2_I and VO_2_I (p < 0.05 for all comparisons)
Boldt *et al.*, 1996 [46]	28	Trauma of ISS > 15	20% albumin vs 10% HES 200/0.5 to 12–16 mm Hg target CVP, PCWP or both	Maximum platelet aggregation declined in both groups (p < 0.05)
Boldt *et al.*, 1996 [48]	28	Trauma of ISS > 15	20% albumin vs 10% HES 200/0.5 to 10–15 mm Hg target PCWP	Vasopressin decreased in HES 200/0.5 but not albumin group (p < 0.05)
Boldt *et al.*, 1998 [49]	150	Trauma of ISS > 15	20% albumin vs 10% HES 200/0.5 to 12–15 mm Hg target PCWP	PaO_2_/FiO_2 _increased by HES 200/0.5 but not albumin (p < 0.05); higher cardiac index, DO_2_I and VO_2_I in HES 200/0.5 group (p < 0.05 for all comparisons); no differences in incidence of renal failure, platelet count, PT or aPTT

aPTT, activated partial thromboplastin time; COP, colloid oncotic pressure; CVP, central venous pressure; DO_2_I, oxygen delivery index; HES, hydroxyethyl starch; ISS, injury severity score; PaO_2_/FiO_2_, ratio of partial pressure of arterial oxygen to fraction of inspired oxygen; PCWP, pulmonary capillary wedge pressure; PT, prothrombin time; VO_2_I, oxygen consumption index.

**Table 2:** Randomized clinical trials of hyperoncotic albumin in sepsis

**Trial**	**n**	**Indication**	**Regimen**	**Results**
Boldt *et al.*, 1995 [44]	30	Sepsis after major surgery	20% albumin vs 10% HES 200/0.5 to 12–16 mm Hg target CVP, PCWP or both	Plasma thrombomodulin increased in albumin group and remained unchanged in HES 200/0.5 group (p < 0.05); plasma protein C among HES 200/0.5 recipients increased on days 4 and 5 without corresponding change in albumin group (p < 0.05)
Boldt *et al.*, 1996 [45]	30	Sepsis secondary to major general surgery	20% albumin vs 10% HES 200/0.5 to 12–18 mm Hg target PCWP	HES 200/0.5 but not albumin increased cardiac index, RVEF, PaO_2_/FiO_2_, DO_2_I and VO_2_I and decreased SVRI (p < 0.05 for all comparisons); pH_i _decreased in albumin but not HES 200/0.5 group (p < 0.05)
Boldt *et al.*, 1996 [46]	28	Sepsis after major surgery	20% albumin vs 10% HES 200/0.5 to 12–16 mm Hg target CVP, PCWP or both	Maximum platelet aggregation declined in both groups (p < 0.05)
Boldt *et al.*, 1996 [47]	42	Sepsis secondary to major surgery	20% albumin vs 6% HES 200/0.5 vs pentoxyfylline (300 mg bolus plus 1.4 mg/kg/h continuous infusion)	Circulating sELAM-1 and sICAM-1 concentrations reduced by HES 200/0.5 compared with albumin (p < 0.05 for both comparisons)
Boldt *et al.*, 1996 [48]	28	Sepsis secondary to major surgery	20% albumin vs 10% HES 200/0.5 to 10–15 mm Hg target PCWP	Vasopressin, endothelin-1, norepinephrine and 6-keto-prostaglandin F_1a _decreased and pH_i _increased in HES 200/0.5 but not albumin group (p < 0.05 for all comparisons); ANP increased by albumin but not HES 200/0.5 (p < 0.05)
Boldt *et al.*, 1998 [49]	150	Postoperative sepsis	20% albumin vs 10% HES 200/0.5 to 12–15 mm Hg target PCWP	PaO_2_/FiO_2 _increased and lactate decreased by HES 200/0.5 but not albumin (p < 0.05 for both comparisons); higher cardiac index, DO_2_I and VO_2_I in HES 200/0.5 group (p < 0.05 for all comparisons); no differences in incidence of renal failure, platelet count, PT or aPTT
Palumbo *et al.*, 2006 [59]	20	Severe sepsis	20% albumin vs 6% HES 130/0.4	PCWP of 15–18 mm Hg successfully maintained by both colloids throughout the 5-day study period; temperature, MAP, pulmonary artery pressure, CVP, heart rate and urine output remained stable in both groups; HES increased cardiac index and several oxygenation parameters and decreased APACHE II score

ANP, atrial natriuretic peptide; APACHE, Acute Physiology and Chronic Health Evaluation; aPTT, activated partial thromboplastin time; CVP, central venous pressure; DO_2_I, oxygen delivery index; HES, hydroxyethyl starch; MAP, mean arterial pressure; PaO_2_/FiO_2_, ratio of partial pressure of arterial oxygen to fraction of inspired oxygen; PCWP, pulmonary capillary wedge pressure; pH_i_, gastric intramucosal pH; PT, prothrombin time; RVEF, right ventricular ejection fraction; sELAM-1, soluble endothelial leukocyte adhesion molecule-1; sICAM-1, soluble intercellular adhesion molecule-1; SVRI, systemic vascular resistance index; VO_2_I, oxygen consumption index.

**Table 3:** Randomized clinical trials of hyperoncotic albumin in other indications

**Trial**	**n**	**Indication**	**Regimen**	**Results**
Liver disease				
Gentilini *et al.*, 1999 [51]	126	Cirrhosis and refractory ascites	Inpatient treatment with 12.5 g/day 25% albumin plus diuretics vs diuretics alone	90.5% cumulative treatment response rate in group receiving albumin vs 74.7% in control group (p < 0.05); shorter hospital stay (p < 0.05) in group receiving albumin (20 vs 24 days) resulting in 59% cost savings; no survival difference
Sort *et al.*, 1999 [53]	126	Cirrhosis with ascites and spontaneous bacterial peritonitis	1.5 g/kg 20% albumin within 6 h of diagnosis and 1 g/kg on day 3 vs no albumin; intravenous cefotaxime in both groups	Renal impairment in 33% of control group vs 10% of albumin recipients (p = 0.002); 29% hospital mortality in control group vs 10% of group receiving albumin (p = 0.01); 41% and 22% mortality, respectively, by 3 months of follow-up (p = 0.03)
Fernández *et al.*, 2005 [58]	20	Cirrhosis and spontaneous bacterial peritonitis	20% albumin vs 6% HES 200/0.5, both administered at 1.5 g/kg after baseline measurements and 1.0 g/kg on day 3	Albumin increased mean arterial pressure and decreased plasma renin activity; no improvements in circulatory function in patients receiving HES; 4 of 10 HES recipients developed spontaneous bacterial peritonitis-induced circulatory dysfunction or renal failure, whereas neither complication occurred in any of the 10 patients receiving albumin
High-risk neonates				
McMurray *et al.*, 1948 [35]	33	Premature infants with low birth weight	3 ml 25 g/dl albumin injected per pound body weight 1–2 times weekly vs no albumin	8.5 days shorter mean time to regain birth weight in infants receiving albumin (p = 0.02) and significantly fewer illnesses
Greenough *et al.*, 1993 [42]	30	Ventilator-dependent ill pre-term infants	5 ml/kg 20% albumin vs placebo	Albumin reduced edema based on weight loss (p < 0.01), whereas control group gained weight (p < 0.05); 27% reduction in inspired oxygen requirement after albumin treatment (p < 0.05) with no change in control group
Gürkan *et al.*, 2001 [56]	18	Newborns with asphyxia and brain edema	0.5 g/kg 20% albumin vs routine fluid	Higher modified Apgar score in group receiving albumin after 24 h (p < 0.001) with difference persisting 8 days; cerebral edema reduced in greater proportion of albumin than control group as judged by head ultrasound; 28% shorter hospital stay in albumin-treated group (p < 0.01)
Brain injury				
Goslinga *et al.*, 1992 [40]	300	Acute ischemic stroke	Normovolemic hemodilution with 20% albumin vs crystalloids	In subgroup with normal hematocrit accounting for two-thirds of study population, mortality and disability at 3 months significantly lower among albumin recipients
Tomita *et al.*, 1994 [43]	18	Closed head injury	High-oncotic-pressure therapy with 25% albumin and furosemide vs normal-oncotic-pressure therapy	Recovery with minimal or no neurological deficit in patients of high-oncotic-pressure therapy group; persistent vegetative state or death in 30% of patients receiving normal-oncotic-pressure therapy
Intradialytic hypotension				
van der Sande *et al.*, 1999 [54]	10	Crossover trial of stable dialysis patients	20% albumin vs 10% HES 200/0.5 vs saline, in conjunction with ultrafiltration and hemodialysis	Greater decrease in blood volume with saline than other fluids (p < 0.05)
van der Sande *et al.*, 2000 [55]	9	Crossover trial of cardiac-compromised dialysis patients	100 ml of 20% albumin vs 10% HES 200/0.5 vs 3% hypertonic saline, in conjunction with ultrafiltration and hemodialysis	Greater intradialytic reductions in systolic blood pressure (p < 0.05) and blood volume (p < 0.05) with hypertonic saline than either albumin or HES
Nephrotic syndrome				
Kosnadi *et al.*, 1987 [38]	24	Children with nephrotic syndrome	20% albumin + furosemide + prednisone vs human plasma + furosemide + prednisone vs prednisone alone	Diuresis earlier with albumin + furosemide + prednisone vs prednisone alone (p = 0.011) and percent body weight loss greater (p < 0.01)
Fliser *et al.*, 1999 [50]	9	Double-blind, placebo-controlled crossover trial in patients with nephrotic syndrome on standardized salt intake	200 ml 20% albumin + 0.9% NaCl sham infusion vs 200 ml 20% albumin + 60 mg furosemide vs 60 mg furosemide + sham infusion of 200 ml H_2_O	Urinary volume and sodium excretion higher by 20% (p < 0.05 and p < 0.01, respectively) during first 8 h with albumin + furosemide than furosemide alone
Na *et al.*, 2001 [57]	7	Crossover trial in patients with nephrotic syndrome	100 ml 20% albumin vs 5% dextrose followed by 160 mg of furosemide	Albumin potentiated the diuretic effect of furosemide

HES, hydroxyethyl starch.

Evaluated study endpoints included survival in three trials [40,51,53], morbidity in six [35,40,43,53,56,58], major organ function in six [41,42,49,56,58,59], length of stay in two [51,56], and costs of care in one [51]. Among additional endpoints assessed were hemodynamics in seven trials [45,48,49,52,54,55,59], major organ edema in two [39,56], whole body edema in two [38,42], coagulation function in four [39,44,46,49], colloid oncotic pressure (COP) in two [36,37], diuretic responsiveness in two [50,57], and inflammatory markers in one [47].

### Trial quality

Four trials (16%) were blinded [41,50,53,56], and the remainder unblinded. Allocation concealment was adequate in four trials (16%) [50,51,53,58] and inadequate or unclear in the rest.

### Surgery

Based on randomized clinical trial data from surgical patients summarized in Table 1, hyperoncotic albumin more effectively maintained colloid oncotic pressure than control regimens in both cardiac [37] and non-cardiac surgery [36]. Compared with HES, hyperoncotic albumin better preserved renal function [41] and coagulation [39]. A greater increase in cardiac output was demonstrated after administration of hyperoncotic albumin than saline to the same right atrial pressure target [52]. Hyperoncotic albumin was superior to both HES and crystalloid in preventing intestinal edema during abdominal surgery [39].

### Trauma

In contrast, among the five randomized comparisons in trauma described by Boldt *et al. *(Table 1) no apparent clinical advantages of hyperoncotic albumin over HES were apparent in three reports [44,46,48]. In the other two reports [45,49], cardiac index and oxygenation were increased by HES relative to albumin.

### Sepsis

Similarly, in sepsis (Table 2) higher cardiac index and oxygenation after HES than albumin administration were observed in several trials [45,49,59]. Additionally, HES maintained higher gastric intramucosal pH than hyperoncotic albumin in two trials [45,48] and improved Acute Physiology and Chronic Health Evaluation II score in one [59].

### Liver disease

In cirrhotic patients with refractory ascites (Table 3), hyperoncotic albumin increased the treatment response rate, shortened hospital stay and reduced costs of care [51]. In patients developing spontaneous bacterial peritonitis, hyperoncotic albumin diminished the incidence of renal impairment [53]. Unlike HES 200/0.5 (molecular weight/molar substitution ratio), hyperoncotic albumin improved circulatory function of patients with spontaneous bacterial peritonitis [58].

### High-risk neonates

In randomized trials of high-risk premature infants (Table 3), hyperoncotic albumin reduced the frequency of illnesses, lessened whole body edema and improved respiratory function [35,42]. Apgar scores were higher, the frequency of cerebral edema lower and hospital stay shorter after hyperoncotic albumin administration in newborns with asphyxia and brain edema [56].

### Brain injury

Hyperoncotic albumin reduced disability at 3 months in patients with acute ischemic stroke and normal hematocrit (Table 3) [40]. Use of hyperoncotic albumin to maintain high oncotic pressure also led to more favorable outcomes in a randomized trial of patients with closed head injury [43]. All patients receiving high-oncotic-pressure therapy recovered with minimal or no neurological deficit, whereas 30% of the control group remained in a vegetative state or died.

### Intradialytic hypotension

In two randomized crossover trials (Table 3), hyperoncotic albumin was more effective than either saline [54] or hypertonic saline [55] in averting blood volume declines. Systolic hypotension was also reduced by hyperoncotic albumin compared with saline [55].

### Nephrotic syndrome

Hyperoncotic albumin was investigated in three randomized trials of nephrotic syndrome (Table 3). Hyperoncotic albumin with concomitant furosemide accelerated diuresis and promoted body weight loss [38]. In addition, hyperoncotic albumin potentiated both the diuretic and natriuretic effects of furosemide [50,57].

### Survival

**Figure 2:**
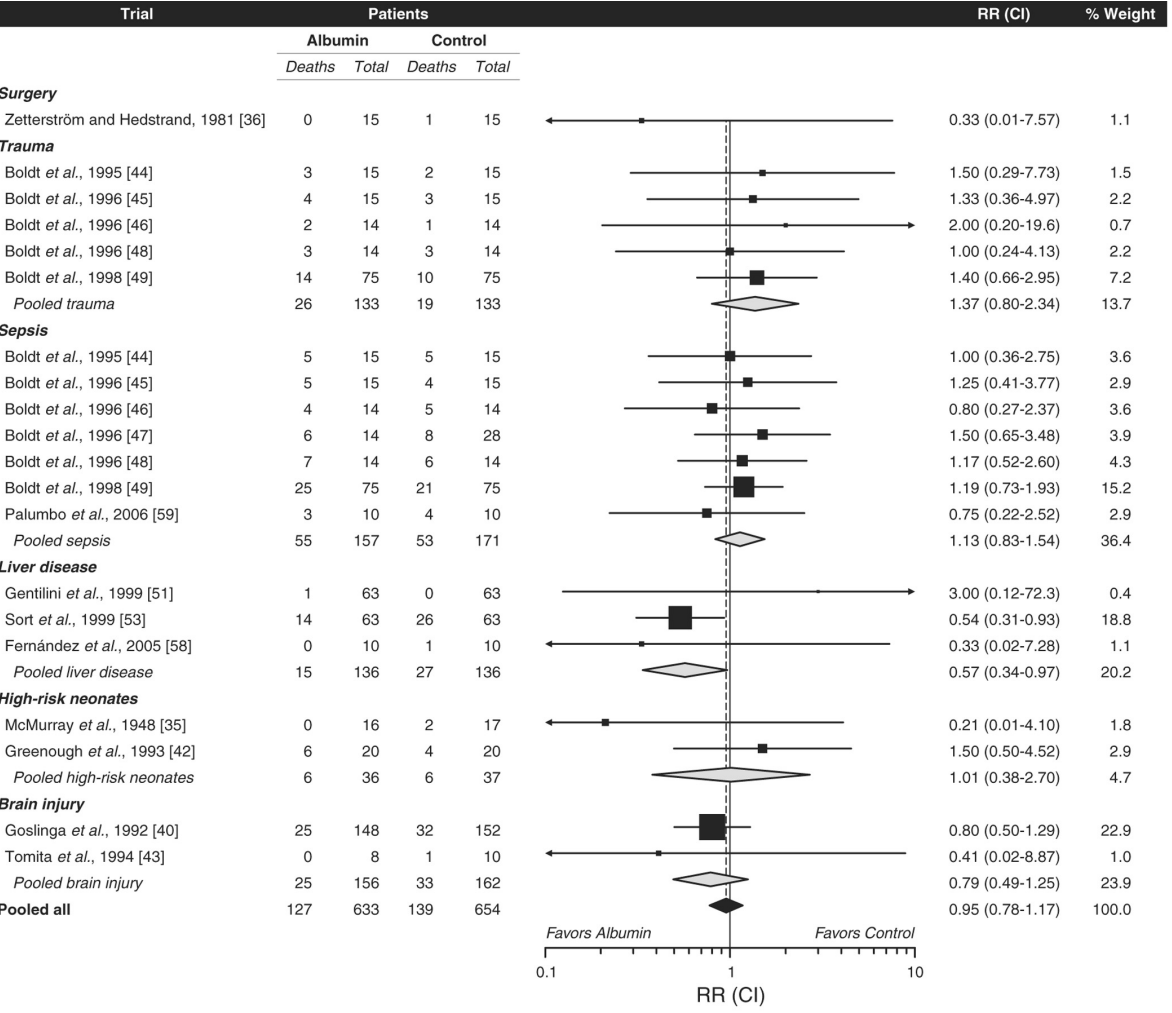
Quantitative meta-analysis of survival in hyperoncotic albumin compared with control group. Data points are scaled in proportion to meta-analytic weight. CI, 95% confidence interval; RR, relative risk.

Mortality data were available for 24 randomized comparisons. The median duration of follow-up was 5 days (IQR, 5 to 13 days). Three trials [40,51,53] were specifically designed to assess survival as a primary study endpoint. No patient died in 4 of the 24 comparisons [37,39,41,56]. The remaining 20 comparisons with at least one death were included in a quantitative meta-analysis of survival (Figure 2). Total mortality in the 20 comparisons (Figure 2) was 266/1,287 (21%). In the meta-analysis, there was no evidence of either heterogeneity (p = 0.86) or publication bias (p = 0.87). The observed statistical power of the meta-analysis was 98% to detect a 35% reduction in relative mortality risk by hyperoncotic albumin (RR 0.65) and 82% to demonstrate a 35% relative mortality risk increase (RR 1.35). No significant overall effect of hyperoncotic albumin on survival was detected (RR 0.95; CI 0.78 to 1.17).

## Discussion

This is the first systematic review exclusively focused on hyperoncotic albumin. With this focus, variability due to potential differences in the effects of 4 to 5% vs 20 to 25% albumin is eliminated.

The included randomized clinical trials supplied evidence of clinical benefits such as reduction in morbidity in high-risk neonates [35] and brain injury [40], avoidance of major organ edema in surgery [39] and high-risk neonates [56] and preservation of renal function in surgery [41] and liver disease [53]. These benefits in the randomized trials are consistent with extensive evidence from non-randomized clinical investigations, as discussed below.

### Limitations

The included randomized trials addressed diverse clinical indications for hyperoncotic albumin, and the number of trials focusing on any single indication was, in most instances, relatively small. Within any particular indication, furthermore, no more than two trials evaluated clinically relevant endpoints of the same type. Consequently, firm conclusions about clinical benefit in defined indications are difficult to draw. Another limitation was the variety of control regimens. Treatment effects of hyperoncotic albumin might differ, for instance, in comparison to crystalloid vs colloid control fluid.

The available data in the categories of trauma and of sepsis were predominantly furnished by a single group of investigators, and 10 of the 11 randomized comparisons from this group were performed using the same trial design (Tables 1 and 2). The generalizability of those results is uncertain, and further trials are needed in those categories.

In the survival meta-analysis, the short median follow-up of 5 days may account partly or entirely for the lack of overall effect. Follow-up of 30 days or longer is typical for outcomes trials. Even at 30 days survival, differences may be substantially underestimated compared with those observed at 90 days [60]. In all three included trials designed to assess survival, the follow-up period was at least 90 days [40,51,53].

As in previous systematic reviews of albumin administration [26,27], one of the control regimens represented among the trials in this review was no albumin, accounting for six of the included trials (24%). No albumin as the designated control regimen did not necessarily mean no volume expansion, however. In one surgery trial [36] the no-albumin control group received a cumulative mean volume of 24 l non-colloid fluids over the course of the 5-day study. In two trials of high-risk neonates, the no albumin control regimen consisted of routine fluid [56] or 5 ml/kg infant maintenance fluid [42]. No albumin control group volume expansion, if any, in three other trials was unspecified [35,51,53]. If control patients in any of those three trials did not receive volume expanders, then observed differences between the hyperoncotic albumin and control arms might have reflected volume expansion rather than a specific effect of hyperoncotic albumin *per se*. In that scenario, it is possible that an alternative fluid could exert effects similar to those observed with hyperoncotic albumin.

These limitations highlight the need for further trials. There is consistent evidence that hyperoncotic albumin has the capacity to reduce edema; however, the impact of edema reduction on outcomes requires further investigation. Short follow-up has been a deficiency of most hyperoncotic albumin trials to date, and future trials with longer follow-up are essential to assess outcomes. Especially in the areas of surgery, trauma and sepsis existing data need to be supplemented by additional trials. Lastly, standardized and well-specified control regimens will be important in future trials so that clearer inferences can be drawn concerning the specific effects of hyperoncotic albumin vs those of particular alternative fluids or generic volume expansion.

### Small-volume resuscitation

A small volume can be administered more rapidly, and so the period during which the patient remains at increased risk of poor outcome can be commensurately reduced. In the pre-hospital setting, hyperoncotic albumin could accelerate hemodynamic stabilization and transport to hospital. Another advantage in the pre-hospital setting is the portability of small hyperoncotic albumin volumes.

In surgical patients, small-volume hyperoncotic albumin resuscitation may simplify fluid management by maintaining a stable hemodynamic state. In a retrospective study of 28 patients undergoing pelvic exenterations for gynecological malignancies, recipients of 25% albumin required fewer boluses of fluid (p < 0.01), electrolyte (p < 0.01) and diuretic (p < 0.01) and could be started sooner on central hyperalimentation (p < 0.05) than the group receiving crystalloid [61].

Speed of resuscitation may also be of the essence in rapidly progressing conditions such as spontaneous bacterial peritonitis [53] and in the urgent care of high-risk neonates [20]. In pre-term infants with respiratory distress syndrome, intravenous administration of 1 g/kg 25% albumin over a 10 min period increased blood volume (p < 0.0005) and mean arterial blood pressure (p < 0.05) within 10 min after infusion was completed [20]. Albumin also significantly augmented glomerular filtration in that study, as indicated by increased creatinine clearance (p < 0.005).

### Edema

Formation of edema serves as a harbinger of organ failure [62] and may, depending upon the tissues affected, lead to impairments in gas exchange, myocardial compliance, neurocognitive function, gut barrier competence, nutrient absorption and wound healing. Pulmonary edema, for instance, is associated with prolonged ventilator dependence and intensive care unit stay [63]. Furthermore, positive fluid balance appears to be predictive of poorer survival in sepsis [64]. Encephalopathy may occur at least as frequently as other forms of organ damage during sepsis [65]. Contributors to septic encephalopathy are reduced cerebral blood flow and oxygen extraction by the brain and cerebral edema [65]. While edema may be adequately tolerated by many patients, such as young serious trauma victims, more grave consequences may be faced by elderly or frail patients in whom edema may retard oxygen delivery to the lungs, myocardium and brain.

The impact of cerebral edema on outcome after subarachnoid hemorrhage (SAH) was investigated in 374 patients [66]. In a multivariate analysis assessing 16 significant univariate predictors, cerebral edema demonstrated by computed tomography was an independent risk factor both for death (odds ratio 2.5; CI 1.1 to 5.6) and for either death or severe disability (odds ratio 2.5; CI 1.2 to 5.4). Severe disability was defined as a modified Rankin scale score of > 3. These observations support a causal role for cerebral edema in producing poor outcomes and prompted the investigators to conclude: 'Critical care management strategies that minimize edema formation after SAH may improve outcome' [66]. Similarly, in a prospective study of 113 non-traumatic SAH patients evaluated by comprehensive neuropsychological testing at 3 months, cerebral edema was an independent risk factor for cognitive impairment [67].

Hyperoncotic albumin may be of particular clinical value in edematous states such as those encountered in liver disease, high-risk neonates, brain injury and nephrotic syndrome (Table 3). While edema may pose problems in sepsis as well, this review suggested advantages of HES over albumin in sepsis, e.g., higher cardiac index and oxygenation. However, follow-up in all the included sepsis trials was only 5 days. With follow-up of 30 days or longer HES has been shown in randomized trials to increase the incidence of acute renal failure among patients with severe sepsis or septic shock [60,68]. Hyperoncotic albumin displays protective effects on the kidney [41,53] and hence would likely offer a safer fluid management alternative than HES in sepsis.

The randomized trial evidence showing the capacity of hyperoncotic albumin to reduce edema is supplemented by non-randomized clinical studies and animal models in the areas of high-risk neonates, brain injury and nephrotic syndrome. Among 10 normotensive premature infants with idiopathic respiratory distress syndrome, 20% albumin reduced body weight (p < 0.01), indicating lessened edema, and improved urine output (p < 0.05) within 6 h [69].

Decreased cerebral edema after hyperoncotic albumin administration was shown in one included randomized trial [56]. Reduction in cerebral edema and other sequelae of brain injury by hyperoncotic albumin has also been repeatedly demonstrated in other studies. In a non-randomized trial of 22 patients with putaminal hemorrhage, 50 to 100 ml/day of 25% albumin with concomitant furosemide significantly reduced brain edema as assessed by computed tomography [70]. No albumin recipient died or remained in a vegetative state compared with 27% of crystalloid recipients. In a cohort study of 10 patients with elevated intracranial pressure and brain edema, infusion of 2 g/kg 25% albumin over 60 min produced a significant and long-lasting (≥ 9 h) decline in intracerebral pressure [71]. Among hemorrhagic stroke patients, rapid infusion of 50 ml 20% albumin promptly and significantly reversed electroencephalographic abnormalities [72]. The randomized, double-blinded, placebo-controlled multi-center Albumin in Acute Stroke (ALIAS) clinical trial funded by the US National Institutes with a target enrollment of 1,800 patients is evaluating the neuroprotective effects of 25% albumin as compared with saline [73].

According to experimental cerebral ischemia studies, hyperoncotic albumin can reduce infarct volume and edema, augment cortical perfusion and improve functional outcome [74,75]. In head trauma models, hyperoncotic albumin increased neurological score and reduced brain tissue edema and damage [76-78].

All three included randomized trials in nephrotic syndrome [38,50,57] indicated the ability of hyperoncotic to promote diuresis, and one of these trials [38] also provided direct evidence of edema reduction. In a non-randomized study of 14 severely edematous children with minimal change nephrotic syndrome, 20% albumin infused at an albumin dose of 0.5 g/kg over 1 h followed by furosemide reduced pretibial edema and body weight within 1 h (p < 0.05) [79]. These effects persisted for at least 24 h (p < 0.005).

### Anti-inflammatory activity

Starling forces may be sufficient to explain the effectiveness of hyperoncotic albumin for small-volume resuscitation and edema reduction. However, additional mechanisms such as anti-inflammatory and antioxidant activity may contribute to the clinical benefits of hyperoncotic albumin. Furthermore, at least some of these mechanisms are specific to hyperoncotic rather than 4 to 5% albumin. Thus, in an *in vitro *study of blood specimens from 10 healthy adult human volunteers, dilution with HES 450/0.7 produced a dose-dependent increase in neutrophil activation to 19-fold the baseline level [80]. The increase after dilution with various crystalloids was to 13- to 19-fold the baseline level and with 5% albumin twofold. In contrast, there was no evidence of neutrophil activation by 25% albumin.

### Antioxidant effects

The reduced thiol moiety on cysteine 34 of albumin can play a direct antioxidant role. In a randomized clinical trial of 20 patients with acute lung injury due to trauma, pneumonia, sepsis and other insults, repeated administration of 25% albumin elevated plasma thiol concentration (p = 0.0001) and total antioxidant capacity (p = 0.033) compared with saline placebo [81].

### Safety

The capacity of hyperoncotic albumin to shift fluid from the interstitium to the intravascular space may be viewed as a two-edged sword, depending upon the hydration state of the patient. In edematous states, such fluid shifts may be desirable. Conversely, hyperoncotic albumin should probably be avoided in patients with severe dehydration [3].

High doses of hyperoncotic albumin could potentially increase pulmonary edema and capillary leak. In one widely cited randomized trial of Lucas *et al. *[82], patients receiving very high hyperoncotic albumin doses (1,142 g) and total fluid volumes (44.9 l) displayed impairment in pulmonary function. Commentators have ascribed that result to fluid overload rather than hyperoncotic albumin *per se*, however [83,84]. The trial of Lucas *et al. *was not included in the present review because albumin was administered with the aim of elevating serum albumin concentration rather than expanding intravascular volume, and indeed the attained central venous pressure (mean 18.6 cm H_2_O) was supranormal.

In a dose-escalation study of 25% albumin treatment in 82 patients with acute ischemic stroke, mild or moderate pulmonary edema was observed in 13% of the patients [85]. This adverse effect was readily managed with diuretics, and 25% albumin doses up to 2 g/kg could be tolerated without major dose-limiting complications.

None of the trials included in the present review or in a prior systematic review of both hyperoncotic and 4 to 5% albumin [28] has indicated harmful effects attributable to albumin. A Cochrane meta-analysis encompassing both 4 to 5% and 20 to 25% solutions suggested increased mortality among albumin recipients (RR 1.7) [26]. That finding could not be replicated either in the SAFE trial [25] or a more comprehensive subsequent meta-analysis [27]. In the present survival meta-analysis, which was adequately powered to detect a relative mortality risk increase only half as great as that reported by the Cochrane investigators, there was no evidence of poorer survival among hyperoncotic albumin recipients.

## Conclusion

Small-volume resuscitation with hyperoncotic albumin can speed the stabilization of the patient and alleviate potentially harmful edema. Nevertheless, the clinical benefits of hyperoncotic albumin remain incompletely characterized. Further trials are needed, especially in the areas of surgery, trauma and sepsis.

## Key messages

Hyperoncotic 20 to 25% albumin solutions are suitable for small-volume resuscitation.

In a systematic review of 25 randomized clinical trials, several benefits of hyperoncotic albumin were documented such as decreases in morbidity, renal impairment and edema.

There was no evidence of deleterious effects due to hyperoncotic albumin.

Overall survival was not affected by hyperoncotic albumin (pooled relative risk, 0.95; 95% confidence interval 0.78–1.17).

Further clinical trials of hyperoncotic albumin are warranted.

## Abbreviations

ANP = atrial natriuretic peptide; APACHE = Acute Physiology and Chronic Health Evaluation; apt = activated partial thromboplastin time; CI = confidence interval; COP = colloid oncotic pressure; CVP = central venous pressure; DO_2_I = oxygen delivery index; HES = hydroxyethyl starch; IQR = interquartile range; ISS = injury severity score; MAP = mean arterial pressure; PaO_2_/FiO_2 _= ratio of partial pressure of arterial oxygen to fraction of inspired oxygen; PCWP = pulmonary capillary wedge pressure; pH_i _= gastric intramucosal pH; PT = prothrombin time; RL = Ringer's lactate; RR = relative risk; RVEF = right ventricular ejection fraction; sELAM-1 = soluble endothelial leucocyte adhesion molecule-1; sICAM-1 = soluble intercellular adhesion molecule-1; SVRI = systemic vascular resistance index; VO_2_I = oxygen consumption index.

## Competing interests

The Clinic of Anesthesiology, Ludwig-Maximilians University Munich, Munich, Germany, with which MJ, DC, PC and MR are affiliated, was the recipient of an unrestricted research grant from CSL Behring, Marburg, Germany supporting investigations of interactions between colloids and the endothelial glycocalyx. That work resulted in three original contributions. The grant was not linked to any specific research goals or to manuscript approval by CSL Behring. MJ has received lecture honoraria from B. Braun, Melsungen, Germany and Fresenius Kabi, Bad Homburg, Germany. MMW has received past unrestricted research grant support from CSL Behring and, prior to 2003, the Plasma Protein Therapeutics Association, Annapolis, Maryland, USA. BFB has no competing interests to declare.

## Authors' contributions

All authors participated in the design of the study. MJ, MMW and MR extracted data and drafted the manuscript. All authors revised the article and read and approved the final manuscript.
